# Supplementary material for: Therapeutic Effects of Hypoxic and Pro-Inflammatory Priming of Mesenchymal Stem Cell-Derived Extracellular Vesicles in Inflammatory Arthritis
Source: Int J Mol Sci. 2021 Dec 23;23(1):126. doi: 10.3390/ijms23010126 (PMC8745583; doi:10.3390/ijms23010126)
Supplement: Supplementary file 1 [file ijms-23-00126-s001.zip › ijms-1505459-supplementary.pdf]

International Journal of
Molecular Sciences

MDPI

Supplementary Materials (to be published online)

Figure S1

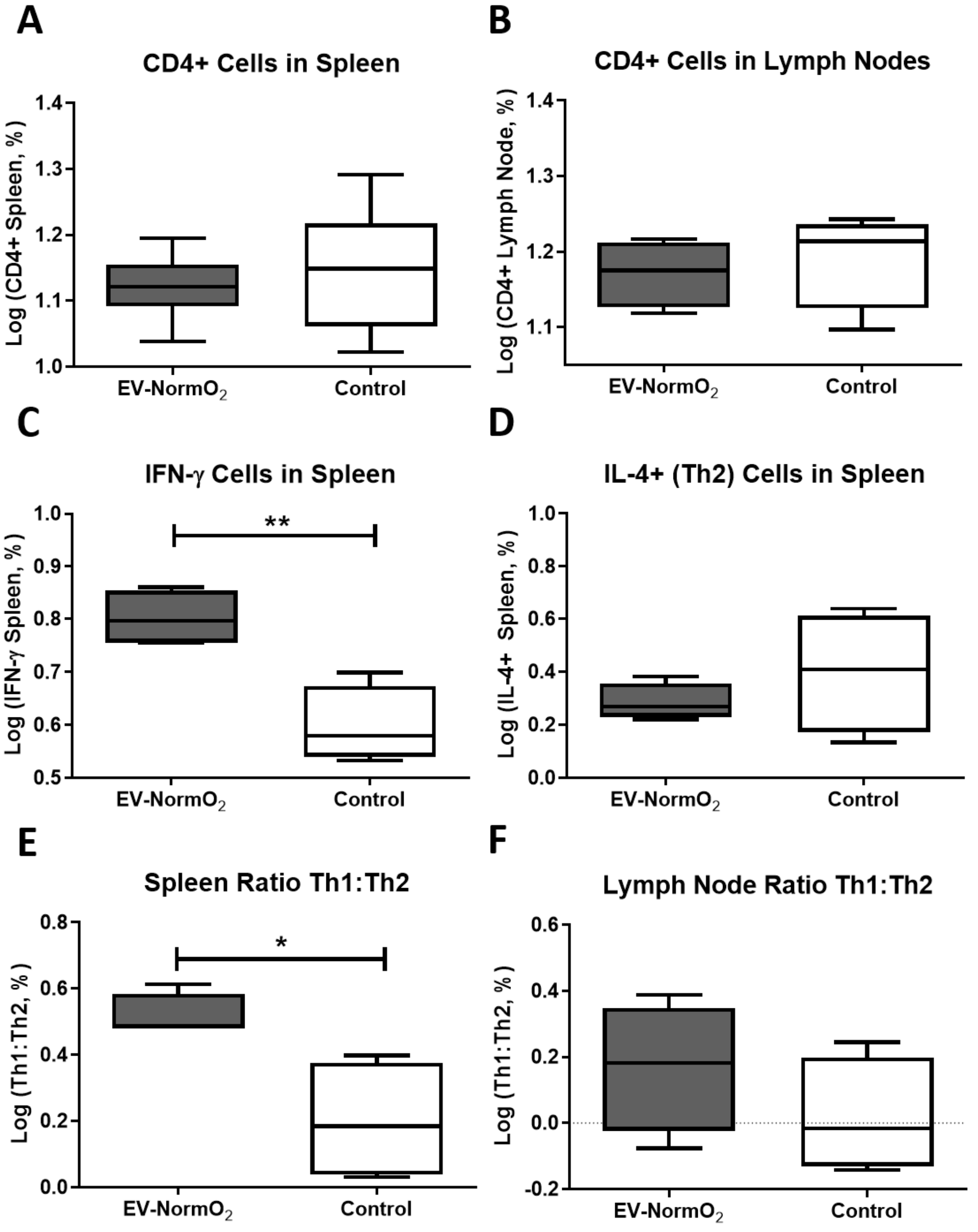

Figure S1 – Outcomes of intracellular staining for T cell polarisation analysis. Intracellular staining for FACS analysis of IFN-γ, IL4 and IL17a in CD4+ T cells from EV-NormO2, EV-depleted CM-MSC or PBS control spleen and lymph node tissues. (A) EV-NormO2 treatments maintained lower CD4+ cell presence in spleen tissue compared to EV-depleted MSC-CM treatments but this effect was not replicated in (B) lymph node cells. (C/D) No changes were observed in IFN- expressing Th1 or IL-4 expressing

Th2 cells with (E/F) no resulting differences seen in the Th1:Th2 ratio in either spleen or lymph node tissues (n=4, *p<0.05; **p<0.01; ***p<0.001)(1 Way ANOVA with Bonferroni Multiple Comparisons Test post hoc using log-transformed data.

Figure S2

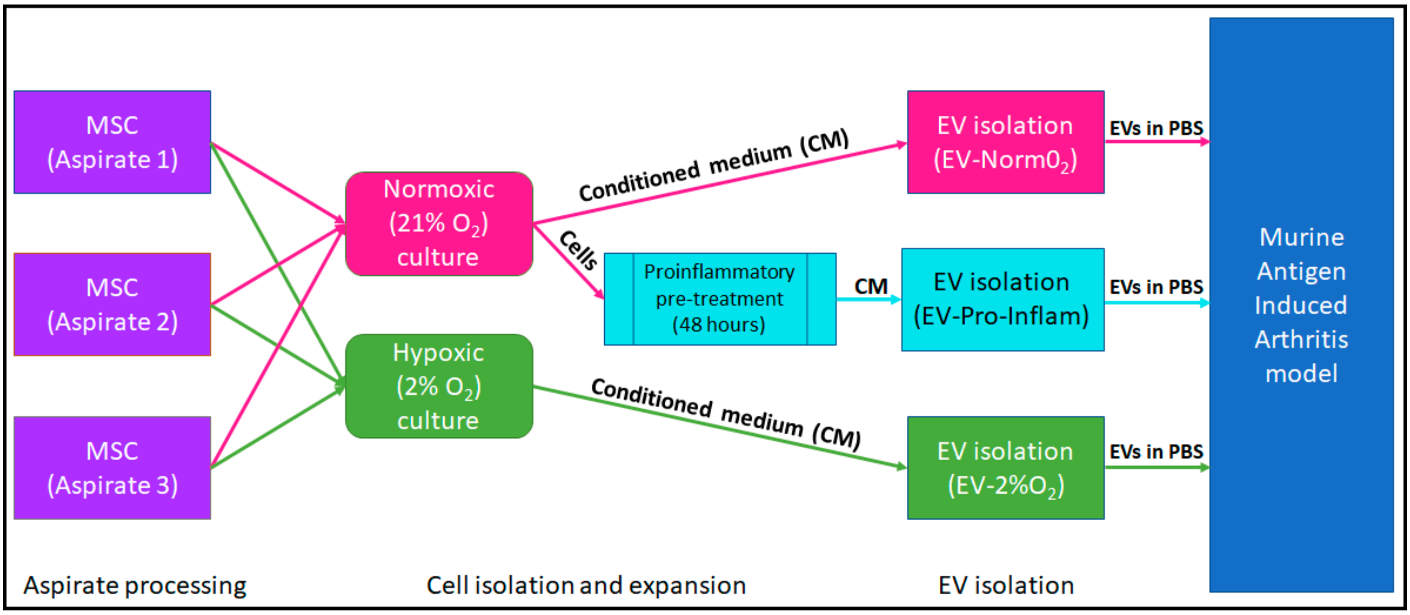

Figure S2 – MSC aspirate processing for the production of extracellular vesicle (EVs) treatments and application into the antigen induced arthritis (AIA) model. Three independent aspirate samples sourced commercially (Lonza) were split to culture in normoxic (21% O2) and hypoxic (2% O2) conditions and conditioned medium (CM) collected from each. Normoxically produced MSCs were also exposed to proinflammatory cytokine cocktail as detailed and CM collected. All CM collections were individually processed to extract EVs which were then applied as therapeutic treatments into the AIA model.
